# The influence of patient-centeredness on patient safety perception among inpatients

**DOI:** 10.1371/journal.pone.0246928

**Published:** 2021-02-12

**Authors:** Nahee Choi, Jinhee Kim, Hyunlye Kim

**Affiliations:** 1 Office of Quality Improvement, Chosun University Hospital, Gwangju, South Korea; 2 Department of Nursing, College of Medicine, Chosun University, Gwangju, South Korea; University of New South Wales, AUSTRALIA

## Abstract

**Purpose:**

This study investigated the influence of patient-centeredness on patient safety perception among inpatients, with particular focus on the relationships between subfactors of patient-centeredness and patient safety perception.

**Methods:**

Data were collected from 122 inpatients in a university hospital from September 24 to October 8, 2019. Patient-centeredness was evaluated using the Patient-Centeredness Assessment Scale; patient safety perception was evaluated using the Korean version of the Patient Safety Perception Scale. Multiple linear regression analysis was conducted using SPSS for Windows 24.0.

**Results:**

Average patient-centeredness score among inpatients was 77.14 ± 12.64 (range 0–100), and average patient safety perception score was 99.24 ± 15.90 (range 24–120). Patient-centeredness influenced patient safety perception (R^2^ = 70%, F = 27.75, *p* < .001). With respect to subfactors of patient safety perception, the medical team’s activities to ensure safety was affected by the general treatment process and overall evaluation of patient-centeredness (R^2^ = 54%, F = 13.14, *p* < .001); patient safety practice was influenced only by the general treatment process (R^2^ = 39%, F = 7.02, *p* < .001); and trust in the medical system was affected by nurses’ service, the general treatment process, and the hospital environment (R^2^ = 44%, F = 8.49, *p* < .001).

**Conclusions:**

To enhance patient safety perception, strategies should seek to strengthen patient-centeredness and its related subfactors, particularly the general treatment process, the hospital environment, and nurses’ service.

## Introduction

Patient-centeredness (PC) in medical care refers to supporting patients and their guardians, reflecting patients’ priorities and wishes in clinical decision-making, and placing patients at the center of the relationship between patient and medical team [1]. Since early 2000, the World Health Organization, the Institute of Medicine, and the Organization for Economic Cooperation and Development have defined PC as a core aspect of quality healthcare [2–5]. Accordingly, many countries have come to consider patient-centered healthcare an essential factor when evaluating healthcare quality and the performance of healthcare systems [6].

To induce the improvement of healthcare quality through the evaluation of the patient experiences in South Korea, the Health Insurance Review & Assessment Service (HIRA) had improved PC developed in a preceding study [2], conducted patient experience evaluation in tertiary general and general hospitals, and released the results to the public [7]. Furthermore, the HIRA announced that patient experience assessment results would be applied as a new evaluation index for healthcare quality beginning in 2020. Accordingly, medical institutions have been trying to improve inpatients’ satisfaction with the patient-centered healthcare services they receive.

Patient safety is a topic of focus worldwide, with patient safety incidents estimated to rank tenth among causes of death and permanent damage [8]. High-income countries experience patient safety incidents in one out of 10 people, whereas mid- to low-income countries reported 134 million adverse event cases and 2.6 million deaths [8]. In 2013, preventable patient safety incidents were the second highest cause of death in South Korea, resulting in approximately 19,800 deaths [9]. Counseling for medical disputes also rapidly increased from 26,256 cases in 2012 to 42,268 cases in 2014 [9]. In response, the Korean government enacted the Patient Safety Act in July 2016, implementing systematic approaches and management to prevent patient safety incidents [10]. According to Article 5 of this Act, all patients have the right to receive safe health care services, and patients and their guardians must participate in patient safety activities. Thus, it is vital for patients to participate actively in patient safety practices as the center of their own treatment process. To ensure safe healthcare service, patients themselves should recognize patient safety issues and actively engage in safety-related practices [11, 12].

Patient safety perception refers to the level of perception that a patient receiving medical care views patient safety [11]. Patients are an important source of information which helps improve healthcare through reducing harm that can be prevented. Patients can proactively identify potential risks to safety in hospitals setting through patient measure of safety [13]. Therefore, to improve the quality of medical care through the prevention of patient safety accidents, it is necessary to improve patient-centered patient safety awareness [12].

To prevent patient safety incidents, the United States has implemented a program that promotes patient and family participation in patient safety activities [14, 15]. Looking at this in detail, to enhance patient safety and healthcare quality, the Agency for Healthcare Research and Quality (AHRQ) has developed and distributed a *Guide to Patient and Family Engagement in Hospital Quality and Safety*, which emphasizes active participation of patients and their families in the treatment process [14]. Since 2002, the Joint Commission has run a “Speak Up” campaign to engage patients and their families in patient safety [15]. In hospitals, “speaking up” means that patients directly express concerns to the medical team when they feel that the team’s activities are dangerous or aspects of the treatment process are omitted [16]. This campaign is intended to help patients and their guardians actively participate in treatment processes, emphasizing that the patients’ considerations are central in the treatment process.

In South Korea, patient safety issues were discussed intensively after introduction of the Healthcare Accreditation System in 2010. However, exploration of patient safety has focused on healthcare providers [17, 18], a trend also seen in other countries [19, 20]. Although studies of patient safety with a focus on patients have recently been initiated [11, 13, 20–22], few studies have directly investigated patient safety perception (PSP) with a focus on patients, or the effects of PC on patients’ PSP.

Therefore, this study aimed to identify PC experiences and PSP among inpatients, and to explore the effects of PC on PSP, thereby providing fundamental data for development of strategies to foster patient safety culture from patients’ perspective. To obtain detailed data, the study particularly focused on the relationship between subfactors of PC and PSP.

### Objective

This study aimed to identify the effects of inpatients’ PC experience on their PSP. Four specific objectives were identified. First, to identify PC and PSP experienced by inpatients; second, to identify differences in PC and PSP depending on general characteristics; third, to investigate the relationship between PC and PSP among inpatients; and finally, to examine the effects of PC on inpatients’ PSP.

## Methods

### Study design

The present study adopted a descriptive correlational design to identify PC experience and PSP among inpatients and to investigate the effects of PC on PSP.

### Participants and data collection

The study’s participants were patients admitted to a university hospital for at least three days, scheduled for discharge on the day of their response to the questionnaires. All participants were provided with a full explanation of the study and its objectives and gave their consent to participate. Minimum hospital stay was set to three days so that participants’ responses would reflect sufficient admission experiences. Additional inclusion criteria were being 18 years or older; having no history of psychiatric issues; being able to communicate and respond to the questionnaire; and being scheduled for discharge from general wards, excluding special departments (e.g., emergency room, intensive care units, or emergency wards).

Sample size was determined using G*POWER version 3.1. With the assumption of a two-tailed test for multiple regression analysis, an alpha (α) level of .05, power (1-β) of .80, medium effect size (*f*²) of .15, and 10 predictors for multiple regression analysis, a sample size of at least 118 participants was calculated. Considering a 10% drop-out rate, data were collected from 130 participants.

Data were collected in 13 general wards in a university hospital between September 24 and October 8, 2019. Before data were collected, the purpose of the study and the contents of the questionnaire were explained to the head of the nursing department of the hospital, and with their cooperation, a list of patients scheduled to be discharged the next day was provided every evening during the data collection period. After receiving the list of patients to be discharged that day, the researcher visited these patients in the morning and explained the purpose and content of the study. For patients who voluntarily consented to participate in the study, data were collected using a self-report questionnaire. A total of 130 questionnaires were distributed and collected (100% response rate). After excluding 8 questionnaires with incomplete responses, a total of 122 questionnaires (93.8%) were included in the final analysis.

### Measurement instruments

#### Patient-Centeredness Assessment Scale

PC was assessed using 24 items from the Patient-Centeredness Assessment Scale developed by Do et al. [2] measuring seven subfactors (nurses’ service, 4 items; physicians’ service, 4 items; general treatment process, 8 items; hospital environment, 2 items; guarantee of rights, 3 items; fair treatment, 1 item; and overall evaluation, 2 items). This tool is available to the public. The general treatment process subfactor, which contains the most items, includes explanation of administration/examination/treatment, participation in treatment processes, pain control, consideration, emotional support, and description of treatment plan after discharge. Items were rated on a 4-point Likert scale from 1 (*Never*) to 4 (*Always*). To calculate scores, the ratings were converted as follows: 1 = 0 points, 2 = 33 points, 3 = 67 points, and 4 = 100 points. The item in the guarantee of rights subfactor was rated either “Yes” or “No”; to calculate scores, a response of “Yes” received 100 points, and a response of “No” received 0 points. Items in the overall evaluation subfactor were rated on a 11-point scale from 0 (*worst*) to 10 (*best*). To calculate scores, the ratings were converted as follows: 0 = 0 points, 1 = 10 points, 2 = 20 points, 3 = 30 points, 4 = 40 points, 5 = 50 points, 6 = 60 points, 7 = 70 points, 8 = 80 points, 9 = 90 points, and 10 = 100 points. Scores for each subfactor were calculated as the arithmetic mean of the items included in the subfactor, and total PC scores were calculated as the arithmetic mean of the scores in each subfactor [2], with higher scores indicating a higher level of PC experienced. Cronbach’s α was .88 for the overall scale and .62 to .85 for the subfactors at the time of development; in this study, Cronbach’s α was .81 for the overall scale and .52 to .83 for the subfactors.

#### Patient Safety Perception Scale (Korean version)

PSP was assessed using the Korean version of the Patient Safety Perception Scale, with approval from the original author [11]. This tool includes 24 items measuring three subfactors (activities to ensure safety, 10 items; patient’s safety practice, 10 items; and trust in the medical system, 4 items). Each is rated on a 5-point Likert scale from 1 (*strongly disagree*) to 5 (*strongly agree*), with higher scores indicating higher PSP. Cronbach’s α was .93 for the overall scale and .62 to .85 for the subfactors in the original study. In this study, Cronbach’s α was .86 for the overall scale and .90 to .93 for the subfactors.

### Ethical considerations

The study protocol was approved by the Institutional Review Board (IRB) of the university hospital where data were to be collected (IRB No. 2019-07-010-006). Before beginning the study, permission was granted by the head of the nursing department and cooperation of those who were in charge. All participants were provided with an explanation of the study’s objectives. They were assured that their participation was voluntary and there was no penalty for not participating. They were informed of their options to withdraw from or suspend participation in the study at any time, and of their anonymity and the preservation and disposal of the study’s data. The survey was implemented after obtaining participants’ written consent. Participants spent about 20 minutes to complete the questionnaire and were offered a small gift as thanks for their participation.

### Data analysis

All data analyses were conducted using SPSS for Windows 24.0. Participants’ general characteristics were expressed as frequency and percentage. PC and PSP scores were expressed as mean ± standard deviation. Differences in overall PC and PSP as well as subfactors of PSP (activities to ensure safety, patient’s safety practice, and trust in the medical system) according to general characteristics were analyzed based on independent *t*-tests or one-way ANOVA. When homogeneity of variance was assumed in the post-hoc test, Fisher’s LSD test was performed; when homogeneity of variance was not assumed, the Games-Howell test was performed. Pearson’s correlation coefficients were calculated to investigate the relationships between the PC subfactors, including overall PC, and the PSP subfactors, including overall PSP. Finally, multiple regression analysis was performed to investigate the effects of inpatients’ PC experience on their PSP, and the effects of the subfactors of PC on the subfactors of PSP.

## Results

### Participants’ characteristics

The study’s participants included more men (54.1%) than women (45.9%), and their mean age was 53.72 ± 18.29 years, with most participants being 60 years or older (45.1%). Participants with college graduation or higher (39.3%) were the most common, and those with spouses (63.9%) were more than those without. The most common guardian of the patients was their spouses (47.5%), and the primary caregivers during the patients’ hospital admission were also spouses (36.1%). The median length of hospital stay was 8 days (range: 3–42), and more patients were admitted through the emergency room (59.8%) than through outpatient departments (40.2%). More patients were admitted to the department of surgery (59.8%) than the department of internal medicine (40.2%), and the number of most frequent hospitalizations within the last 12 months, including this one, was once or twice (34.4% each). The most common subjective health status of participants was moderate (47.5%) (Table 1).

**Table 1:** Participants’ characteristics and difference in patient-centeredness by participants’ characteristics (N = 122).

Variables	Categories	N (%)	Patient-Centeredness	t/F	*p*
			M±SD		
**Gender**	Male	66 (54.1)	78.28±12.69	1.08	.283
	Female	56 (45.9)	75.80±12.56		
**Age (year)**	≤39	32 (26.2)	74.30±12.43	1.21	.301
	40–59	35 (28.7)	77.39±14.21		
	60≤	55 (45.1)	78.65±11.62		
**Education**	≤Middle School	42 (34.4)	78.15±10.75	0.35	.702
	High School	32 (26.2)	77.57±14.27		
	College≤	48 (39.3)	75.97±13.18		
**Marital Status**	Partnered	78 (63.9)	77.73±12.78	0.68	.499
	Single	44 (36.1)	76.11±12.46		
**Guardian**	Spouse	58 (47.5)	77.08±13.92	2.45	.067
	Parents or Child	51 (41.8)	75.08±11.61		
	Other Family Member	9 (7.4)	85.38±6.56		
	None	4 (3.3)	85.88±4.65		
**Primary Caregiver**	Spouse	44 (36.1)	76.1±14.73	2.16	.078
	Parents or Child	41 (33.6)	76.16±11.60		
	Other Family Member	10 (8.2)	84.90±7.11		
	Employed Caregiver	11 (9.0)	71.48±12.35		
	None	16 (13.1)	81.40±9.32		
**Length of Stay**	3~7 Days	54 (44.3)	78.42±12.23	0.61	.546
	8~14 Days	46 (37.7)	76.63±13.63		
	15 Days≤	22 (18.0)	75.07±11.64		
**Hospitalization Route**	Emergency Room	73 (59.8)	76.09±13.06	-1.12	.263
	Outpatient	49 (40.2)	78.71±11.94		
**Medical Department**	Internal Medicine	49 (40.2)	77.02±12.15	-0.09	.931
	Surgery	73 (59.8)	77.23±13.04		
**Hospitalizations Within the Last 12 Months**	1	42 (34.4)	75.38±13.80	1.36	.260
	2	42 (34.4)	76.45±11.50		
	3≤	38 (31.1)	79.87±12.37		
**Health Status**	Unhealthy	26 (21.3)	78.56±13.18	1.06	.351
	Moderate	58 (47.5)	75.40±13.84		
	Healthy	38 (31.1)	78.83±10.04		

### Experience of patient-centeredness and patient safety perception among inpatients

The average overall PC score among the participants was 77.14 ± 12.64. By subfactor, average scores were 86.54 ± 13.10 for nurses’ service, 76.78 ± 17.08 for physicians’ service, 81.73 ± 16.39 for the general treatment process (including explanation of administration/examination/treatment, participation in treatment processes, pain control, and other), 70.77 ± 23.99 for the hospital environment, 53.28 ± 23.37 for guarantee of rights, 83.61 ± 26.50 for fair treatment, and 73.55 ± 15.69 for overall evaluation of their present hospital admission (Table 2).

**Table 2:** Level of patient-centeredness and patient safety perception among inpatients (N = 122).

Variables	Range	M±SD	Min	Max
**Patient-Centeredness**	0~100	77.14±12.64	40.6	97.8
Service of Nurses	0~100	86.54±13.10	50.0	100.0
Service of Doctors	0~100	76.78±17.08	25.0	100.0
General Treatment Process	0~100	81.73±16.39	34.7	100.0
Hospital Environment	0~100	70.77±23.99	0.0	100.0
Ensuring of Patient Rights	0~100	53.28±23.37	0.0	100.0
Fair Treatment	0~100	83.61±26.50	0.0	100.0
Overall Evaluation	0~100	73.55±15.69	5.0	100.0
**Patient Safety Perception**	24~120	99.24±15.90	40.6	97.8
Activities to Ensure Safety	10~50	41.36±6.97	13.0	50.0
Patient’s Safety Practice	10~50	40.57±7.11	11.0	50.0
Trust of the Medical System	4~20	17.31±3.03	4.0	20.0

Participants’ average overall PSP score was 99.24 ± 15.90. By subfactor, average scores were 41.36 ± 6.97 for activities to ensure safety, 40.57 ± 7.11 for patient’s safety practice, and 17.31 ± 3.03 for trust in the medical system (Table 2).

### Differences in patient-centeredness, patient safety perception, and subfactors depending on participants’ characteristics

No statistically significant differences in PC experience were identified according to participants’ general characteristics (Table 1).

A statistically significant difference in PSP was found according to guardian (F = 4.22, *p* = .007). Because homogeneity of variance was not assumed in the post-hoc test (F = 4.61, *p* = .004), the Games-Howell test was performed. PSP was significantly higher when patients’ guardians were family members such as relatives and siblings (110.78 ± 6.40) than when patients’ guardians were their spouse (100.22 ± 13.16) or parents or children (94.98 ± 18.61). PSP was also significantly higher when patients had no guardian (113.25 ± 6.65) than when parents or children acted as a guardian (94.98 ± 18.61) (Table 3).

**Table 3:** Difference in patient safety perception and its sub-categories by participants’ characteristics (N = 122).

Variables	Categories	N (%)	Patient Safety Perception			Activities to Ensure Safety			Patient’s Safety Practice			Trust of the Medical System		
			M±SD	t/F	*p*	M±SD	t/F	*p*	M±SD	t/F	*p*	M±SD	t/F	*p*
**Gender**	Male	66 (54.1)	99.95±14.68	0.54	.591	41.80±6.54	0.76	.449	40.52±6.71	-0.08	.933	17.64±2.79	1.29	.200
	Female	56 (45.9)	98.39±17.33			40.84±7.48			40.63±7.61			16.93±3.29		
**Age (year)**	≤39	32 (26.2)	96.53±16.89	0.86	.427	40.03±7.52	0.92	.400	39.97±7.46	0.62	.542	16.53±3.05	1.45	.239
	40–59	35 (28.7)	101.63±12.75			42.31±5.99			41.69±5.73			17.63±2.34		
	60≤	55 (45.1)	99.29±17.10			41.53±7.23			40.20±7.72			17.56±3.37		
**Education**	≤Middle School	42 (34.4)	98.55±17.78	0.06	.938	41.43±7.52	0.01	.990	39.62±8.03	0.57	.568	17.50±3.44	0.23	.795
	High School	32 (26.2)	99.81±15.73			41.44±7.53			40.97±6.83			17.41±2.69		
	College≤	48 (39.3)	99.46±14.54			41.25±6.21			41.13±6.47			17.08±2.92		
**Marital Status**	Partnered	78 (63.9)	100.04±13.78	0.74	.461	41.67±6.35	0.64	.521	40.83±6.21	0.55	.582	17.54±2.66	1.10	.273
	Single	44 (36.1)	97.82±19.19			40.82±8.01			40.09±8.53			16.91±3.61		
**Guardian**	Spouse	58 (47.5)^a^	100.22±13.16	4.22	.007* (a,b<c; b<d)	41.76±6.03	3.00	.034^†^ (b<c)	41.02±6.16	3.95	.010* (a,b< c,d)	17.45±2.60	4.36	.006* (a,b<c;b<d)
	Parents or Child	51 (41.8)^b^	94.98±18.61			39.75±7.96			38.71±8.10			16.53±3.51		
	Other Family Member	9 (7.4)^c^	110.78±6.40			45.56±4.56			45.33±3.43			19.89±0.33		
	None	4 (3.3)^d^	113.25±6.65			46.75±4.72			47.00±2.45			19.50±1.00		
**Primary Caregiver**	Spouse	44 (36.1)	100.73±12.99	2.43	.051	42.20±6.08	2.24	.069	40.93±6.15	1.86	.122	17.59±2.62	2.42	.052
	Parents or Child	41 (33.6)	97.44±18.52			40.61±7.59			39.93±7.95			16.90±3.56		
	Other Family Member	10 (8.2)	109.40±7.68			45.50±4.65			44.60±3.63			19.30±1.34		
	Employed Caregiver	11 (9.0)	89.36±13.53			37.18±6.16			36.64±7.46			15.55±2.94		
	None	16 (13.1)	100.19±18.10			41.25±8.15			41.38±7.83			17.56±2.90		
**Length of Stay**	3~7 Days	54 (44.3)	99.54±14.38	0.10	.906	41.35±6.66	0.19	.823	40.70±6.06	0.02	.977	17.48±2.63	0.24	.783
	8~14 Days	46 (37.7)	98.46±19.46			41.00±8.10			40.39±8.89			17.07±3.73		
	15 Days≤	22 (18.0)	100.14±11.02			42.14±5.17			40.59±5.40			17.41±2.36		
**Hospitalization Route**	Emergency Room	73 (59.8)	97.99±16.61	-1.06	.291	41.36±7.08	-0.01	.993	39.60±7.59	-1.84	.068	17.03±3.18	-1.27	.208
	Outpatient	49 (40.2)	101.10±14.76			41.37±6.89			42.00±6.12			17.73±2.79		
**Medical Department**	Internal Medicine	49 (40.2)	98.49±16.99	-0.42	.672	41.29±7.10	-0.10	.923	39.96±7.67	-0.77	.443	17.24±3.19	-0.20	.844
	Surgery	73 (59.8)	99.74±15.23			41.41±6.94			40.97±6.73			17.36±2.95		
**Hospitalizations Within the Last 12 Months**	1	42 (34.4)	101.10±13.13	0.43	.650	42.07±5.69	0.33	.720	41.36±6.12	0.42	.657	17.67±2.74	0.58	.563
	2	42 (34.4)	98.24±16.05			40.95±6.85			40.33±7.20			16.95±3.02		
	3≤	38 (31.1)	98.29±18.57			41.03±8.39			39.95±8.08			17.32±3.38		
**Health Status**	Unhealthy	26 (21.3)	99.88±19.25	1.42	.246	41.73±8.33	1.74	.180	40.88±7.72	0.64	.527	17.27±3.82	1.85	.162
	Moderate	58 (47.5)	96.88±16.02			40.21±7.37			39.83±6.95			16.84±3.04		
	Healthy	38 (31.1)	102.39±12.73			42.87±4.89			41.47±7.00			18.05±2.27		

* Post-hoc comparison = Games-Howell;

^†^Post-hoc comparison = Fisher’s LSD

The PSP subfactor of activities to ensure safety also showed statistically significant differences depending on guardian (F = 3.00, *p* = .034). Because the post-hoc test resulted in an assumption of homogeneity of variance (F = 2.30, *p* = .081), Fisher’s LSD test was performed. Activities to ensure safety was found to be statistically significantly higher when guardians were family members such as relatives and siblings (45.56 ± 4.56) than when they were parents or children (39.75 ± 7.96). The subfactor of patient’s safety practice also differed significantly depending on guardian (F = 3.95, *p* = .010). Because the post-hoc test resulted in no assumption of homogeneity of variance (F = 3.67, *p* = .014), the Games-Howell test was performed. Patient’s safety practice was significantly higher when guardians were family members such as relatives and siblings (45.33 ± 3.43) or when patients had no guardian (47.00 ± 2.45) than when guardians were the patient’s spouse (41.02 ± 6.16) or parents or children (38.71 ± 8.10). Finally, trust in the medical system also differed significantly depending on guardian (F = 4.36, *p* = .006). The post-hoc test resulted in no assumption of homogeneity of variance (F = 7.04, *p* < .001), so the Games-Howell test was performed. Trust in the medical system was found to be significantly higher when guardians were family members such as relatives and siblings (19.89 ± 0.33) than when they were the patient’s spouse (17.45 ± 2.60) or parents or children (16.53 ± 3.51). It was also significantly higher when patients had no guardian (19.50 ± 1.00) than when the guardians were parents or children (16.53 ± 3.51) (Table 3).

### Correlation between patient-centeredness and patient safety perception

Patients’ ratings of PSP and PC were positively correlated (*r* = .61, *p* < .001). The first subfactor of PSP, activities to ensure safety, showed statistically significant positive correlations with all subfactors of PC (all *p*s < .05). The second subfactor, patient safety practice, showed statistically significant positive correlations with nurses’ service, physicians’ service, the general treatment process, the hospital environment, guarantee of rights, and overall evaluation (all *p*s < .05). The final subfactor, trust in the medical system, showed statistically significant positive correlations with nurses’ service, physicians’ service, the general treatment process, the hospital environment, and overall evaluation (all *p*s < .001) (Table 4).

**Table 4:** Correlation between patient-centeredness and patient safety perception (N = 122).

Variables	Patient Safety Perception		Activities to Ensure Safety		Patient’s Safety Practice		Trust of the Medical System	
	r	*p*	r	*p*	r	*p*	r	*P*
**Patient-Centeredness**	.61	< .001	-	-	-	-	-	-
Service of Nurses	-	-	.51	< .001	.37	< .001	.45	< .001
Service of Doctors	-	-	.48	< .001	.39	< .001	.36	< .001
General Treatment Process	-	-	.61	< .001	.53	< .001	.50	< .001
Hospital Environment	-	-	.30	.001	.22	.014	.40	< .001
Ensuring of Patient Rights	-	-	.27	.003	.22	.015	.13	.142
Fair Treatment	-	-	.21	.020	.12	.189	.04	.635
Overall Evaluation	-	-	.50	< .001	.32	< .001	.38	< .001

### Factors influencing patient safety perception

Multiple regression analysis was conducted after excluding one outlier with an absolute value of 3 or more using case-by-case diagnosis to identify factors influencing PSP and its subfactors (activities to ensure safety, patient’s safety practice, and trust in the medical system) (Table 5). The independent variables in the regression equations were the general characteristics (i.e., guardian) of which statistically significant differences in the level of PSP or PSP subfactors were confirmed, and PC or PC subfactors. The dependent variables were PSP and its subfactors. The dependent variables were PSP and its subfactors. To confirm the satisfaction of the basic assumptions of the regression analysis, the results of linearity, normal distribution, and equidistribution were all satisfied. Additionally, tolerance of the regression equations was .11 to .94 (corresponding to 1.0 or lower) and variance inflation factor (VIF) was 1.06 to 8.83 (corresponding to less than 10), indicating no multicollinearity among the independent variables. The Durbin-Watson statistics ranged from 1.910 to 2.484, indicating no residual autocorrelation in the regression equations, and thus the assumption of the regression analysis was considered satisfactory [23]. The results revealed that PSP was influenced by inpatients’ experience of PC (β = .65, *p* < .001), with an explanatory power (R^2^) of 70% (F = 27.75, *p* < .001). The PSP subfactor of activities to ensure safety was influenced by the PC subfactors of general treatment process (β = .39, *p* < .001) and overall evaluation (β = .20, *p* = .025), with an explanatory power (R^2^) of 54% (F = 13.14, *p* < .001). Patient’s safety practice was influenced by general treatment process (β = .49, *p* < .001), with an explanatory power (R^2^) of 39% (F = 7.02, *p* < .001). Finally, trust in the medical system was influenced by general treatment process (β = .35, *p* = .003), nurses’ service (β = .20, *p* = .044), and hospital environment (β = .21, *p* = .016), with an explanatory power (R^2^) of 44% (F = 8.49, *p* < .001).

**Table 5:** Factors influencing patient safety perceptions (N = 121).

Variables	Patient Safety Perceptions					Activities to Ensure Safety					Patient’s Safety Practice					Trust of the Medical System				
	B	SE	β	t	*p*	B	SE	β	t	*p*	B	SE	β	t	*p*	B	SE	β	t	*p*
**Constant**	49.39	8.58	-	5.75	< .001	13.75	4.04	-	3.40	.001	24.46	4.76	-	5.14	< .001	8.05	1.93	-	4.16	< .001
**Guardian (/ref. Nonexistence)**	-	-	-	-	-	-	-	-	-	-	-	-	-	-	-	-	-	-	-	-
**Spouse**	-6.48	5.52	-.22	-1.17	.243	-2.08	2.48	-.16	-0.84	.404	-3.54	2.92	-.27	-1.22	.227	-0.71	1.19	-.13	-0.60	.551
**Parents or Child**	-8.81	5.57	-.30	-1.58	.117	-2.43	2.48	-.18	-0.98	.330	-4.52	2.92	-.34	-1.55	.124	-0.81	1.19	-.14	-0.69	.494
**Other Family**	-2.11	6.37	-.04	-0.33	.741	-1.42	2.82	-.06	-0.50	.616	-1.93	3.32	-.08	-0.58	.562	0.69	1.35	.06	0.51	.612
**Patient-Centeredness**	0.74	0.08	.65	9.46	< .001	-	-	-	-	-	-	-	-	-	-	-	-	-	-	-
**Service of Nurses**	-	-	-	-	-	0.08	0.04	.17	1.90	.060	0.01	0.05	.03	0.26	.796	0.04	0.02	.20	2.04	.044
**Service of Doctors**	-	-	-	-	-	0.02	0.03	.06	0.75	.454	0.02	0.04	.04	0.41	.686	0.00	0.02	-.02	-0.17	.862
**General Treatment Process**	-	-	-	-	-	0.15	0.04	.39	3.73	< .001	0.20	0.05	.49	4.07	< .001	0.06	0.02	.35	3.01	.003
**Hospital Environment**	-	-	-	-	-	-0.01	0.02	-.03	-0.39	.700	-0.01	0.02	-.03	-0.39	.694	0.02	0.01	.21	2.45	.016
**Ensuring of Patient Rights**	-	-	-	-	-	0.01	0.02	.03	0.38	.702	0.02	0.02	.07	0.87	.384	-0.01	0.01	-.08	-0.93	.354
**Fair Treatment**	-	-	-	-	-	0.03	0.02	.11	1.65	.102	0.01	0.02	.03	0.36	.719	0.00	0.01	-.05	-0.63	.531
**Overall Evaluation**	-	-	-	-	-	0.08	0.04	.20	2.27	.025	0.01	0.04	.02	0.15	.878	0.01	0.02	.06	0.63	.531
	R^2^ = .70, Adj. R^2^ = .47, F = 27.75, *p* < .001, Durbin-Watson = 2.241					R^2^ = .54, Adj. R^2^ = .50, F = 13.14, *p* < .001, Durbin-Watson = 1.910					R^2^ = .39, Adj. R^2^ = .33, F = 7.02, *p* < .001, Durbin-Watson = 2.135					R^2^ = .44, Adj. R^2^ = .38, F = 8.49, *p* < .001, Durbin-Watson = 2.479				

## Discussion

This study identified PC experience and PSP among inpatients as well as the effects of PC on PSP, aiming to provide fundamental data with which to develop strategies to foster patient safety culture from patients’ perspectives.

Average PC experience score among inpatients was 77.14 ± 12.64 (range: 0–100) in the present study, lower than the score (83.94 ± 15.98) reported by the HIRA Patient Experience Assessment [7]. This discrepancy might be attributable to differences in the two studies’ data collection tools (the present study used a modified version of HIRA’s Patient Experience Assessment tool), participants (patients admitted to the hospital for at least three days in the present study vs. patients admitted for at least one day in HIRA’s Patient Experience Assessment), and data collection time (HIRA’s Patient Experience Assessment collected data by telephone after discharge, whereas the present study collected data using a self-administered questionnaire on the day of discharge). Therefore, there are limitations to direct comparison of this study’s results with those of HIRA’s Patient Experience Assessment. The results should be interpreted considering such limitations, and additional research is needed to confirm the present study’s results.

Few existing studies measure and report PC experiences among inpatients. One preceding study involving local diabetes patients used a mail survey to measure the gap between scores for perceived level of chronic care over the previous six months and preferred level of care, and presented this gap as PC level, with higher PC levels relating to a better understanding of disease and less perceived impact of illness [24]. However, there are some limitations in comparing PC level from the previous study with the measurements of the present study. PC is defined as “providing care that is respectful of and responsive to individual patient preferences, need, and values, and ensuring that patient values guide all clinical decisions” [25]; it is a core factor in healthcare quality and the main focus in today’s healthcare field for improving healthcare service quality. However, almost no research on PC experienced by patients has been conducted in the nursing field. Future studies in this area are needed.

Of all PC subfactors, nurses’ service received the highest scores, consistent with a previous study by HIRA [7]. Because nurses provide the most personal patient care at all times, compared to other healthcare staff, they play the most important roles in implementation of PC [26]. Previous research [27] has also indicated that the quality of nurses’ service has a greater effect on patients’ experiences than the quality of physicians’ service. The effects of nurses’ service quality on overall healthcare service should be continuously investigated and evaluated in the nursing field to realize qualitative improvement in healthcare service, the core value of healthcare. Patient-centered care interventions have been shown to improve patients’ knowledge about their health, self-care behavior management skills, satisfaction, and quality of life, and to reduce hospital admissions, readmissions, and length of stay [28, 29]; thus, a patient-centered care intervention to improve nurses’ service quality should be developed and evaluated.

The lowest-scoring PC subfactor in this study was guarantee of rights. This is inconsistent with the HIRA Patient Experience Assessment, which reported physicians’ service, administration, and treatment processes as the lowest-rated subfactors [7]. Future studies should confirm these differences. Patients have the right to receive medical care, to know about their care, to self-determine their treatment, to have their confidentiality protected, to request consulting and mediation, and to have their cultural and religious values or beliefs respected; medical teams should educate inpatients about these patient rights and duties [30]. The healthcare paradigm has been shifting focus from healthcare providers to healthcare consumers (patients). Medical teams should respect patients’ diverse values and beliefs, prioritize patients’ rights and interests during treatment processes, and provide thorough explanations and encouragement so patients can comfortably present their opinions. Such actions should enhance patient safety by enabling patients to actively participate in their treatment processes [12].

Average PSP score among inpatients in this study was 99.24 ± 15.90 (range: 24–120). It is difficult to compare these results with preceding studies because few have investigated PSP among inpatients. Pursuing patient safety refers to preventing accidental or avoidable injuries during medical practice [31]; the aim is to minimize unnecessary healthcare-related risks of damage to an acceptable degree [32]. In other words, medical teams must view cases from the patients’ perspective to ensure that their safety is neither compromised nor at risk [12]. To improve patient safety levels, patients should establish cooperative relationships with their medical teams through active participation and communication [13]. By actively participating in the treatment process and engaging in their own patient safety activities, patients can help ensure that they receive safe healthcare services. However, despite the necessity that patients assume a central role, previous studies on patient safety have focused on healthcare providers as the major actors [20, 33]. Thus, strategies to establish patient safety culture from the patients’ perspective should be discussed.

This study found that PC had a major influence on PSP among inpatients. PC is a new paradigm for realizing patient safety culture by reducing medical errors [34]. To foster patient safety culture, it is important for healthcare consumers (patients) to actively participate in the healthcare process [12, 13]. In other words, to receive safe healthcare services, patients should actively participate in their treatment processes, including establishing an accurate diagnosis, determining and implementing appropriate treatment methods, selecting safe treatments, detecting adverse events, and taking proper measures [12]. To promote this, education programs for current and future healthcare workers should address patient participation and patients’ rights in the healthcare [34]. Future studies should develop and validate the effects of such educational programs.

This study also compared participants’ general characteristics, and correlation and regression analysis were performed to identify PC subfactors with effects on subfactors of PSP among inpatients (activities to ensure safety, patient’s safety practice, and trust in the medical system). The results showed that the general treatment process had an influence on all PSP subfactors. When patients had positive experiences in the general treatment process, all PSP subfactor levels increased. A preceding study [13] indicated that to improve patient safety, it is important for patients to acquire and form knowledge and health beliefs and to establish cooperative relationships with their medical teams through active participation and communications, instead of playing a passive role. The Institute for Patient and Family-Centered Care has proposed respect, dignity, information sharing, participation in healthcare, and cooperation as core factors of PC [35]. Patient safety culture from the patients’ perspective should be established by strengthening these characteristics of PC. Future research should develop and assess specific intervention programs to accomplish this. Strategies for effective development of PC and patient safety culture must enhance patient-centered healthcare culture and strengthen patient safety culture [36].

Nurses’ service, a subfactor of PC, was found to have a positive effect on inpatients’ trust in the medical system. In nursing science, there has been little exploration of the effects of patient-centered nursing on general PSP and trust in the medical system. In the future, interventional studies in the nursing field should be performed to propose detailed ways patient-centered care can help establish patient safety culture from the patient’s perspective.

While most existing studies on patient safety focus on healthcare providers, the present study provides fundamental data for establishing patient-centered patient safety culture by investigating the effects of PC on PSP among patients, the consumers of healthcare services. However, some limitations of the present study should be considered. First, because participants were selected by convenience sampling of patients admitted to a university hospital, the results have limited generalization. Therefore, it is necessary to check the generalizability by confirming the results of this study through repeated studies. Second, although the survey guaranteed participants’ anonymity, it is possible that patients may have answered questions regarding sensitive subjects more positively than their actual experiences were, as they needed to continue using the hospital’s services. The study’s results should be considered and interpreted in light of these aspects.

## Conclusions

This study aimed to provide fundamental data for developing a strategy to foster patient-centered patient safety culture by investigating the effects of PC experienced by inpatients on their PSP. Average PC score was found to be 77.14 ± 12.64, and average PSP score to be 99.24 ± 15.90. Inpatients’ experience of PC had an effect on their PSP. Subfactors of PC were also found to have effects on subfactors of PSP (i.e., the medical team’s activities to ensure safety, patient safety practice, and trust in the medical system): perception of the medical team’s activities to ensure safety was affected by the general treatment process and overall evaluation of PC; patient safety practice was influenced by the general treatment process; and trust in the medical system was affected by nurses’ service, the general treatment process, and the hospital environment. The results of this study can be applied as fundamental data to develop intervention programs to enhance PSP through improvement of PC. Future research should develop and validate the effects of specific intervention programs to establish patient-centered patient safety culture.

## Supporting information

S1 Appendix Questionnaire (Korean version). (PDF)

S2 Appendix Data of questionnaire. (XLSX)
