# Supplementary material for: The influence of patient-centeredness on patient safety perception among inpatients
Source: PLoS One. 2021 Feb 12;16(2):e0246928. doi: 10.1371/journal.pone.0246928 (PMC7880440; doi:10.1371/journal.pone.0246928)
Supplement: S1 Appendix — (PDF) [file pone.0246928.s001.pdf]

S1 Appendix. Questionnaire (Korean version)

◆ 다음은 환자중심성에 대한 내용입니다. 해당되는 항목에 V로 표시해 주시기 바랍니다.

번호	설문 문항	문항 보기
1	담당 간호사는 귀하를 존중하고 예의를 갖추어 대하였습니까?	① 전혀 그렇지 않았다 ② 가끔 그랬다<br>③ 대체로 그랬다 ④ 항상 그랬다
2	담당 간호사는 귀하의 이야기를 주의 깊게 들어주었습니까?	① 전혀 그렇지 않았다 ② 가끔 그랬다<br>③ 대체로 그랬다 ④ 항상 그랬다
3	담당 간호사는 병원생활에 대해 알기 쉽게 설명해 주었습니까?	① 전혀 그렇지 않았다 ② 가끔 그랬다<br>③ 대체로 그랬다 ④ 항상 그랬다
4	담당 간호사는 귀하가 필요로 할 때 의사 또는 업무 담당자에게 연락하는 등 귀하의 요구를 처리하기 위하여 충분히 노력하였습니까?	① 전혀 그렇지 않았다 ② 가끔 그랬다<br>③ 대체로 그랬다 ④ 항상 그랬다
5	담당 의사는 귀하를 존중하고 예의를 갖추어 대하였습니까?	① 전혀 그렇지 않았다 ② 가끔 그랬다<br>③ 대체로 그랬다 ④ 항상 그랬다
6	담당 의사는 귀하의 이야기를 주의 깊게 들어주었습니까?	① 전혀 그렇지 않았다 ② 가끔 그랬다<br>③ 대체로 그랬다 ④ 항상 그랬다
7	귀하는 담당의사의 회진시간 또는 회진시간 변경에 대한 정보를 충분히 제공 받으셨습니까?	① 전혀 그렇지 않았다 ② 가끔 그랬다<br>③ 대체로 그랬다 ④ 항상 그랬다
8	귀하나 보호자가 담당 의사를 만나 이야기 할 기회가 충분히 있었습니까?	① 전혀 그렇지 않았다 ② 별로 그렇지 않았다<br>③ 약간 그랬다 ④ 매우 그랬다
9	의료진들은 투약이나 검사 처치 전에 그에 대한 이유를 충분히 설명해 주었습니까?	① 전혀 그렇지 않았다 ② 가끔 그랬다<br>③ 대체로 그랬다 ④ 항상 그랬다
10	의료진들은 투약이나 검사 처치 후에 생길 수 있는 부작용에 대해 충분히 설명해 주었습니까?	① 전혀 그렇지 않았다 ② 가끔 그랬다<br>③ 대체로 그랬다 ④ 항상 그랬다
11	의료진들은 검사나 치료결정과정에서 귀하의 선호나 의견을 반영하여 선택할 수 있는 기회를 충분히 주었습니까?	① 전혀 그렇지 않았다 ② 가끔 그랬다<br>③ 대체로 그랬다 ④ 항상 그랬다
12	의료진들은 검사나 치료과정에서 신체노출 등 수치감을 느끼지 않도록 충분히 배려하였습니까?	① 전혀 그렇지 않았다 ② 가끔 그랬다<br>③ 대체로 그랬다 ④ 항상 그랬다

번호	설문 문항	문항 보기
13	의료진들은 귀하의 통증을 줄이기 위하여 적절한 조치를 취하였습니까?	① 전혀 그렇지 않았다 ② 가끔 그랬다<br>③ 대체로 그랬다 ④ 항상 그랬다
14	의료진들은 귀하의 질환에 대한 불안감을 줄이기 위하여 충분히 노력하였습니까?	① 전혀 그렇지 않았다 ② 가끔 그랬다<br>③ 대체로 그랬다 ④ 항상 그랬다
15	병원은 전반적으로 청결하였습니까?	① 전혀 그렇지 않았다 ② 가끔 그랬다<br>③ 대체로 그랬다 ④ 항상 그랬다
16	병원은 전반적으로 안전하고 편안한 환경이었습니까?	① 전혀 그렇지 않았다 ② 가끔 그랬다<br>③ 대체로 그랬다 ④ 항상 그랬다
17	입원기간 동안 진료과정 또는 병원생활에 대한 불만을 접수하는 방법에 대한 안내를 받은 적이 있습니까?<br>① 예 ② 아니오	
18	입원기간 동안 진료과정 또는 병원생활에 대한 불만이 있을 경우 문제점이나 불만을 충분히 제기할 수 있었습니까?<br>① 전혀 그렇지 않았다 ② 가끔 그랬다 ③ 대체로 그랬다<br>④ 항상 그랬다 ⑤ 불만이 없었다	
19	의료진들은 검사나 치료결정과정에서 다른 무엇보다 귀하의 이익을 최우선으로 고려하였습니까?	① 전혀 그렇지 않았다 ② 별로 그렇지 않았다<br>③ 약간 그랬다 ④ 매우 그랬다
20	의료진들은 퇴원 후 주의사항에 대해 상세하게 설명해주었습니까?	① 전혀 그렇지 않았다 ② 별로 그렇지 않았다<br>③ 약간 그랬다 ④ 매우 그랬다
21	의료진으로부터 퇴원 이후의 치료 계획에 대한 정보를 제공 받았습니까?	① 전혀 그렇지 않았다 ② 별로 그렇지 않았다<br>③ 약간 그랬다 ④ 매우 그랬다
22	입원기간 동안 다른 환자와 비교했을 때 의료진으로부터 불공평한 대우를 받은 적이 있습니까?	① 전혀 그렇지 않았다 ② 별로 그렇지 않았다<br>③ 약간 그랬다 ④ 매우 그랬다
23	이 병원에서의 입원 경험을 0점에서 10점 사이의 점수로 평가한다면 몇 점을 주시겠습니까? 0점은 가장 나쁜 경우이고 10점은 가장 좋은 경우입니다.<br>0 | 1 | 2 | 3 | 4 | 5 | 6 | 7 | 8 | 9 | 10	
24	만약 가족이나 친구 중에 입원할 일이 생긴다면 이 병원을 이용하도록 추천하시겠습니까?<br>① 절대 추천하지 않을 것이다 ② 추천하지 않을 것이다<br>③ 추천할 것이다 ④ 반드시 추천할 것이다	

◆ 다음은 환자안전인식에 대한 내용입니다. 해당되는 항목에 V로 표시해 주시기 바랍니다.

번호	문 항	매우 그렇지 않다	그렇지 않다	보통 이다	그렇다	매우 그렇다
1	의료진은 안전한 치료 제공을 위해 서로 협력한다.					
2	의료진은 환자안전을 향상시키기 위해 솔선수범하여 노력한다.					
3	의료진은 나의 치료에 대하여 다른 의료진과 정보를 정확하게 공유한다.					
4	의료진은 장비를 능숙하게 사용한다.					
5	의료진은 검사/수술/투약의 부작용 여부를 항상 확인한다.					
6	의료진은 나의 치료에 대하여 정확한 정보를 제공한다.					
7	나의 치료에 필요한 장비는 항상 제대로 작동한다.					
8	의료진은 밤에 수시로 병실 순회를 수행한다.					
9	의료진은 병원에서 발생하는 환자안전문제를 중요하게 여긴다.					
10	의료진은 치료 전에 항상 나의 동의를 구한다.					
11	나는 낙상을 예방하는 방법에 대해 잘 알고 있다.					
12	나는 내가 받은 각종 검사나 시술, 수술의 목적에 대해 알고 있다.					
13	나는 병원에서 수행되는 환자확인 절차에 대해 알고 있다.					
14	나는 나의 치료와 관련된 주의사항을 알고 있다.					
15	나는 나의 치료에 관한 궁금한 사항을 반드시 질문한다.					
16	나는 나에게 투여되는 약물에 대해 잘 알고 있다.					
17	나는 몸에 이상이 있으면 반드시 의사나 간호사에게 알린다.					
18	나는 언제나 환자안전 수칙을 지킨다.					
19	나는 감염예방을 위해 손씻기를 해야 한다는 것을 알고 있다.					
20	나는 나의 건강에 대한 정보를 의료진에게 충분히 말한다.					
21	나는 병원의 의료정보 보안시스템을 신뢰한다.					
22	나는 병원의 의료 장비가 안전하다고 생각한다.					
23	나는 병원의 의료 기술이 안전하다고 생각한다.					
24	나는 의료진을 일반적으로 신뢰한다.					

*안전보장 활동=1~10; 환자의 안전 실천=11~20; 의료시스템 신뢰=21~24.

◆ **귀하의 일반적 특성에 대한 질문입니다. 해당되는 사항을 기재해 주시기 바랍니다.**

1. 귀하의 연령은?　　만 ________세

2. 귀하의 성별은?　①남자　　　　②여자

3. 귀하의 최종학력은?
   ①무학　　②초졸　　③중졸　　④고졸　　⑤대학재학, 대학졸업　　⑥대학원
   * 중퇴이시면 그 전 졸업학력에 V하여 주십시오(예: 고등학교 중퇴인 경우 중졸에 V하여 주십시오.)

4. 배우자가 있습니까?
   ①있다　　　②없다

5. 귀하의 보호자는 누구입니까?
   ①배우자　②부모　③자녀　④기타가족　⑤기타 : ________　⑥없음

6. 이번 입원 기간 동안 귀하의 주간병인은 누구였습니까?
   ①배우자　②부모　③자녀　④기타가족　⑤간병인　⑥기타 : ________　⑦없음

5. 재원 기간 : _____일(입원일 : 201__년 ____월 ____일 ~ 퇴원일 : 201__년 ____월 ____일)

6. 귀하는 응급실을 통해 입원하셨습니까?
   ①예　　　　②아니오

7. 귀하가 입원하신 진료과는 무엇입니까?　__________과

8. 귀하의 주진단명은 무엇입니까?　__________________

9. 최근 1년 동안 몇 번 입원하셨습니까?　__________번

10. 귀하의 건강은 어떻다고 생각하십니까?
   ①매우 좋다　②좋다　③보통이다　④나쁘다　⑤매우 나쁘다

'환자안전'이란 의료 행위로 인해 발생하는 우연한 또는 예방 가능한 손상이 없는 상태를 의미하며, 의료를 제공 받는 환자의 상태, 결과적 측면과 보건의료와 관련된 불필요한 위해의 위험을 수용 가능한 최소한의 수준으로 감소시키는 것입니다. '환자안전활동'이란 환자안전사고의 예방 및 재발 방지를 위하여 행하는 모든 활동을 말합니다.

11. 귀하는 환자안전을 위하여 환자의 환자안전활동이 필요하다고 생각하십니까?
   ①전혀 필요하지 않다　②필요하지 않다　③필요하다　④매우 필요하다　⑤잘 모르겠다
